# Leisure programme trends and shifts amidst the COVID-19 pandemic for adults with disabilities in the City of Cape Town municipality townships, South Africa

**DOI:** 10.4102/ajod.v15i0.1895

**Published:** 2026-08-10

**Authors:** Vhulenda Mukwevho, Makhaya J. Malema, Zeenat Yassin-Burns

**Affiliations:** 1 Department of Sport, Recreation and Exercise Science, Faculty of Community and Health Sciences, University of the Western Cape, Cape Town, South Africa; 2 Department of Child and Family Studies, Faculty of Community Health and Sciences, University of the Western Cape, Cape Town, South Africa; 3 Africa Centre for Inclusion Health Management, Stellenbosch University, Stellenbosch, South Africa

**Keywords:** COVID-19, leisure, leisure programmes, recreation, trends, shift, people with disabilities

## Abstract

**Background:**

The coronavirus disease 2019 (COVID-19) pandemic brought significant disruptions to people’s lives and livelihoods. These disruptions led to changes in people’s mobility, leisure choices and preferences. The need to understand the shifts and emerging trends in leisure programmes for people with disabilities is particularly important in South Africa, where lockdown measures were considered among the strictest and most restrictive.

**Objectives:**

The current study aimed to explore and describe the trends and shifts in leisure programmes for people with disabilities during and after the COVID-19 pandemic in the Western Cape.

**Method:**

The study adopted a descriptive qualitative research approach and utilised semi-structured, open-ended interviews with 12 adults with disabilities. Data were collected until saturation was achieved, and thematic analysis was employed to analyse the data. In accordance with ethics standards, approval was obtained from the University of the Western Cape’s Humanities and Social Sciences Research Ethics Committee.

**Results:**

The key findings revealed five main themes that highlight the disparities experienced by people with disabilities. There was a clear indication that people with disabilities long for leisure participation; however, major barriers need to be broken to ensure smooth participation. People with disabilities still have a strong desire to be involved in leisure programmes, despite being excluded from mainstream activities in their communities.

**Conclusion:**

In conclusion, there is a need for united efforts to ensure that resources are allocated properly, especially during pandemics, given the unique circumstances of people with disabilities.

**Contribution:**

The present study contributes to the literature by emphasising the importance of advocacy for people with disabilities, especially in unprecedented times.

## Introduction

The present study is complementary to a study conducted by Malema et al. (2024), which explored the experiences of leisure time physical activity participation levels and programming needs among people with disabilities. The present study extends the latter study by focusing on leisure trends and shifts in programmes by adults with disabilities. Therefore, participating in leisure activities is considered important for the current study. Leisure in the current study context refers to unscheduled time during which individuals engage in activities that bring feelings of refreshment and amusement (Hurd & Anderson 2023). Malema, Weilbach and Watson (2019) and Parr and Lashua (2004) support this notion, reporting that leisure can be experienced as an activity, free time and state of mind, which offers opportunities for relaxation, self-advocacy and social interaction, and can also be used to relieve stress from daily work and family responsibilities. Leisure activities could include passive activities such as walking, dancing, reading, painting, gardening, cooking, crafting, and woodworking, which can be considered as passive activities (Malema, Weilbach & Watson 2020). Active physical activities can include, but are not limited to, badminton, squash, soccer, and jogging that take place during free time away from work (Hurd & Anderson 2023; Maciel et al. 2024; Malema et al. 2019).

Leisure activities contribute positively to an individual’s development and overall wellbeing (Belošević & Ferić 2022). There was perceived advocacy for increasing calls to participate in leisure activities, particularly amid the coronavirus disease 2019 (COVID-19) pandemic in South Africa. This was driven by the impact of isolation during the COVID-19 era, in which people’s freely chosen leisure programmes and activities were halted. This notion is supported by the Charter for Leisure, which promotes leisure as a basic human right for all individuals (Veal & Sivan 2024). In terms of participation levels in leisure activities, people with disabilities still have low levels of involvement (Allan et al. 2018; Tsai & Fung 2005). This remains concerning given the limitations and marginalisation that people with disabilities face regarding leisure participation. Fiorilli et al. (2021) state that people with disabilities view the rapid disruption of their daily schedules, social interactions, and the opportunity to overcome their limitations through leisure activities as a traumatic occurrence that exceeds their capacity to cope. The trends and shifts that occurred during the COVID-19 pandemic were a paradox, particularly regarding passive and active leisure experiences.

### Leisure for people with disabilities

There are various measures that can promote inclusion, such as ensuring that staff are trained and aware of the needs and support for people with disabilities (Mencap 2019). Access to leisure spaces and opportunities can significantly affect community life for people with disabilities, potentially creating barriers to independence and to the enjoyment of leisure activities (Equality and Human Rights Commission [EHRC] 2017). It must be noted that providing physical access is not limited to wheelchair users; consideration should also be given to improving access for people with a wide range of disabilities (Douglas College 2018). A significant proportion of people with disabilities experience challenges associated with accessing public, commercial, and leisure goods and services (Burns, Watson & Paterson 2013; Papworth Trust 2018).

People with disabilities often require support from family, friends, and coaches, particularly in leisure activities. Families become the first and most consistent source of encouragement. They provide holistic support and are the immediate people who can assist with equipment and exploration of leisure pursuits (Fägerskiöld & Mattsson 2010). Beyond family, especially in physical activity-related programmes, coaches and peers play a critical role: they offer people with disabilities a sense of belonging and help build confidence. Individuals who understand the needs of people with disabilities and provide appropriate support help create welcoming and less intimidating environments. Meaningfully, peer relationships encourage collaboration, teamwork, motivation, and a sense of community for people with disabilities (Chen, Sun & Dai 2017; Svanelöv et al. 2020). Inclusive societies have systems in place to facilitate peer interaction, offering various platforms and methods to promote equal opportunities through policies and infrastructure (Carnemolla, Robinson & Lay 2021). Unfortunately, this level of support is not immediately available within South African communities, which continue to face deep social inequalities.

### Leisure research on COVID-19 in South Africa

The COVID-19 pandemic caused global disruption, leading to social, political, and economic consequences (Lukman et al. 2020). The impact of the pandemic varied across countries, depending on how it was experienced. Additionally, factors such as limited access to immediate healthcare and reduced social events further compounded the situation (Schippers, Ioannidis & Joffe 2022). According to Schippers et al. (2022), the mitigating efforts to limit the spread of the COVID-19 virus had a disproportionate impact on people with disabilities. In South Africa, leisure was not considered a primary sector, which led to strict measures and closure of leisure and tourism spaces and facilities (Young 2020). As a result of global strategies to limit the spread of COVID-19, people turned to home-based leisure activities (Sivan 2020). Young (2020) reported that despite the new area of uncertainties, people embraced family-oriented leisure pursuits. The present study contributes to the literature on this topic by drawing on South African studies on leisure and COVID-19.

The COVID-19 pandemic resulted in both emotional and physical isolation of individuals. Due to restrictions on sports and recreational facilities, leisure spaces, and further movement restrictions to essential services, social life experienced severe disruptions. People became accustomed to changes in their social lives, spending more time indoors on Do-It-Yourself (DIY) tasks, art, and music (Wegner et al. 2022). The recent COVID-19 pandemic has led to increased use of online spaces, where people embrace opportunities to connect and learn from one another. Living in a society where technology encourages people to engage in leisure activities with their gadgets, such as phones and laptops (Lukman et al. 2020), it is evident that during COVID-19, leisure activity trends shifted online. In retrospect, through explorations and innovative means, some people found that these creative activities were more than simply a means to kill time; they provided opportunities for connection for those with close ties to family and friends and served as a lifeline for mental health (Wegner et al. 2022). To preserve their mental wellbeing during the pandemic lockdown, young adults in South Africa also reported a shift towards self-selected indoor activities (Wegner et al. 2022). This change reflects a deeper shift in the understanding and appreciation of leisure that occurred during the pandemic and in the period that followed.

During COVID-19, the world entered a period of uncertainty, and people began exploring ways to continue engaging in leisure activities. As observed and reported by Malema et al. (2021) and Young (2020), reflective studies have reported the state of leisure in South Africa. Malema et al. (2021) reported on online sports and games, which were among the trends that emerged during the COVID-19 pandemic. People embraced online games and sports as a means of mitigating boredom during periods of stricter lockdown. Similarly, Young’s (2020) study focused on observations made by South African’s experiences in how they experienced leisure in their homes during the COVID-19 lockdown. The social dimension of leisure decreased as physical contact connections became challenging, leading many people to turn to online or isolated hobbies that required less social engagement (Lukman et al. 2020). This situation was even worse for people with disabilities, particularly in South Africa’s context of historical inequalities and unequal resource distribution. During the pandemic, people with disabilities encountered challenges related to livelihoods, access to leisure spaces and opportunities for recreation due to the restricted movement imposed by lockdown regulations (Malema et al. 2021, 2024). These regulations meant that people around the world, both with and without disabilities, had to be innovative in finding ways to enjoy their leisure by exploring alternatives to their usual activities.

According to Yang et al. (2025), individuals residing in areas with a high concentration of amenities such as parks, restaurants, and entertainment venues were more inclined to adopt remote work, thereby facilitating deeper integration of leisure into the local environment. While the transformation of leisure patterns is evident, there remains a significant gap in our understanding of the broader and longer-term implications of this shift. As stated by Martínez et al. (2024), the pandemic exacerbated existing inequalities and highlighted critical gaps in access to online recreational, educational, and social opportunities. Shah et al. (2020) argue that physical separation, lockdowns, and masks changed leisure, making it more isolated, home-centred, and digitally mediated. The onset of the COVID-19 pandemic, accompanied by widespread lockdowns, public health concerns, and rapid digital advancements, brought about significant changes in how people devote their leisure time. Activities that once revolved around physical gatherings, travel and shared cultural experiences shifted towards online interactions, home isolation and personalised digital entertainment. The COVID-19 pandemic not only redefined conventional working conditions by accelerating the shift towards remote employment but also disrupted and reorganised established patterns of leisure engagement.

### Theoretical underpinning

**FIGURE 1:**
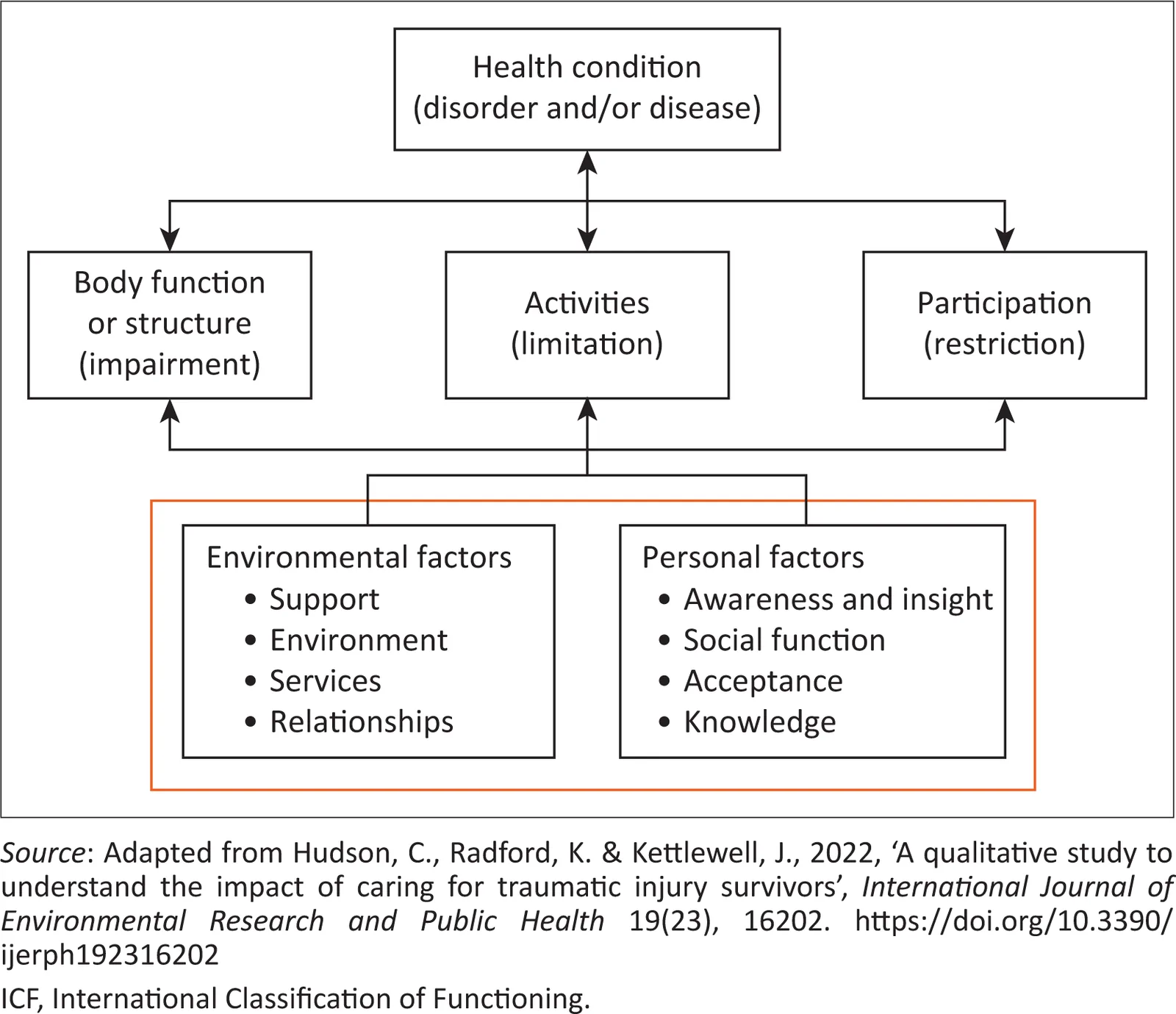
International Classification of Functioning, Disability and Health with expanded environmental and personal factors.

The International Classification of Functioning (ICF) was utilised to theoretically underpin the study. To the knowledge of the researchers, the present study is the first to use the ICF as a framework in the field of leisure for people with disabilities in South African contexts. Malema (2022) in his study on the use of leisure education as a tool for leadership development for youth with disabilities. Additionally, Keats (2019) study explored factors affecting participation in active leisure activities by people with physical disabilities. These two studies, however, did not solely use the ICF as the only framework underpinning their research, unlike the present study. The ICF is a global tool for understanding and classifying health, disability, and functioning (World Health Organization [WHO] 2001, 2023). It recognises that a person’s ability to function and the level of disability are influenced by a combination of their health condition (disability), the environment they live in (e.g. the COVID-19 pandemic), and their personal characteristics (WHO 2001). The present study aligns with the three domains: Health, Environment, and Personal Factors. Figure 1 illustrates the ICF model, which is adopted for the purpose of the current study.

This includes environmental elements, as individuals’ functioning and disabilities occur within a specific context (WHO 2023). The main components of the ICF include bodily functions and structures, activities and participation, environmental factors, and personal factors (Cherney & Carpenter 2022). During the COVID-19 pandemic, societal restrictions and limited access to services further infringed upon the rights and mobility of people with disabilities. Cigman (2010) argued that the use of wheelchairs is not considered a functional limitation when ramps and elevators are built into buildings, reframing wheelchair users as individuals with distinct mobility needs who should be accepted in society.

### Challenges faced by people with disabilities in leisure spaces

People with disabilities have the right to equal opportunities to participate in leisure activities. People with disabilities often face more challenges compared to their able-bodied peers when it comes to participating in sports and recreation. Factors such as social stigma, insufficient support services, and inaccessible environments make it difficult for them to participate (Gomwe, Phiri & Marange 2023; Lindsay Smith et al. 2017). Without adaptive sports programmes or supportive environments, many of these young individuals are left out. This type of exclusion can hinder social and emotional development at a time in their lives when they are shaping their identities. It can also lead to loneliness and low self-esteem (Gomwe et al. 2023; Lindsay Smith et al. 2017). There was a notable shift during the COVID-19 pandemic, particularly in the South African context, due to strict lockdown measures imposed by the government. In public spaces, people were encouraged to wear masks to help reduce virus transmission (South African Government 2020). As a result, people became accustomed to isolation and to limiting their movements and interactions in large crowds (Law et al. 2006). Consequently, online platforms such as TikTok, Instagram, Facebook, and Twitter gained popularity, providing spaces for people to connect (Malema et al. 2021). However, the leisure choices and preferences for people with disabilities remained unclear, despite the availability of virtual platforms as alternatives. This presented a lack of suitable platforms for people with disabilities to participate in during these unique environments. The present study sought to answer the research question: What are the trends and shifts in leisure programmes during and post the COVID-19 pandemic for people with disabilities in the Western Cape? The aim of the study was to explore and describe trends and shifts in leisure participation during and after the COVID-19 pandemic among people with disabilities in the Western Cape.

## Research methods and design

The present study adopted a qualitative research approach using a descriptive design (Creswell & Poth 2016). The present study’s objectives were: (1) to describe the leisure programme trends that occurred during the COVID-19 pandemic for people with disabilities in the Western Cape; and (2) to explore the leisure programme shift that occurred post-COVID-19 pandemic for people with disabilities in the Western Cape. In the present study, the descriptive design is suitable as it allows for an in-depth understanding of the lived experiences of people with disabilities (Campbell & Machado 2013; Shosha 2012).

### Research setting

The research was conducted within the City of Cape Town municipality, in the Western Cape province of South Africa, in the townships Nyanga, Langa, Khayelitsha, and Dunoon. These townships were purposefully selected because the lockdown in townships was experienced differently, mainly due to their overpopulation and under-resourcing, with minimal service delivery. Participants were recruited from non-governmental organisations (NGOs) and community recreation centres that support and advocate for the rights of people with disabilities.

### Data collection procedure

In this study, 12 participants with physical disability who were able to communicate and comprehend information independently to avoid misinterpretation and influence were recruited. Participants with additional disabilities were excluded from the present study because their experiences would have required support from relevant stakeholders, such as sign language interpreters and Braille translators. This support was unavailable to the second researcher during the study process. This approach is a limitation of the present study, and the researchers have made recommendations to address this gap for future researchers. Data collection continued until data saturation was reached (Varpio et al. 2017). After the 10 interviews, no new data emerged; therefore, the researcher conducted an additional two interviews to ensure the rigour of the findings. Purposive sampling was employed to recruit adults with disabilities residing in the chosen locations. The present study was limited to adults with physical disabilities due to mobility limitations, as they were perceived to face the greatest challenges in accessing leisure spaces because of the use of various walking aids. As a non-probability sampling technique, purposive sampling focuses on individuals who have relevant knowledge and lived experience aligned with the research objectives (Nyimbili & Nyimbili 2024). One of the purposive strategies implemented is homogeneous sampling (Campbell et al. 2020).

Homogeneous sampling is a purposive strategy in which participants are selected based on shared characteristics; in this study, having a disability ensured that participants shared key experiences. This targeted approach allows for a deeper exploration of shared experiences within this population (Guest, Bunce & Johnson 2006). The study gathered data through individual interviews with participants following a semi-structured interview guide with open-ended questions (Campbell & Machado 2013). Interviews were conducted either in person (Creswell & Poth 2016). Interview questions were developed based on the literature and refined through a pilot study (Guest et al. 2006). Interviews lasted approximately 30 min and were audio-recorded with participants’ consent and transcribed verbatim by an experienced transcriber. Data were collected at the venues convenient to the participants.

### Data analysis

The thematic analysis framework and guidelines by Braun and Clarke (2006) were used for this study. Thematic analysis is a flexible and widely used qualitative method that allows researchers to identify, analyse and report patterns or themes within data (Braun & Clarke 2006). The process involves several steps, including familiarisation with the data, generating initial codes, searching for themes, reviewing themes, defining and naming themes and producing the final report (Braun & Clarke 2006). Firstly, the interview transcripts were read, and notes were taken during the initial coding process from the transcripts. Each transcript was carefully read and coded to identify recurring ideas and concepts. These codes were then organised into themes that reflected the core issues related to the influence of social support on the participants’ sporting experiences (Braun & Clarke 2006). Secondly, initial codes were formulated and themes identified in line with the interview questions and objectives of the study. Thirdly, the themes were reviewed to identify repetition and grouping the themes to align with the objectives of the study. Themes relevant to the questions were identified and grouped into broad categories after each interview to track which themes were raised. During coding, we referred back to the research question to check our purpose in coding, which is called immersion in the data (Matthews & Kostelis 2011).

### Trustworthiness

Trustworthiness refers to how accurate and credible the study’s data are; it is divided into four categories: Credibility, dependability, transferability and conformability (Schurink, Fouché & De Vos 2011). Credibility in qualitative research refers to the plausibility of the study results, ensuring that they accurately represent what participants express (Shenton 2004). This process was achieved through thorough engagement with participants regarding their experiences and perceptions. To ensure proper representation of their experiences, the researchers checked with the participants to confirm that it accurately reflected their perceptions and experiences through member checking and debriefing sessions. Dependability demonstrates that the research investigation is dependable and reproducible (Graneheim & Lundman 2004). This was achieved by providing a detailed account of each step to ensure that the study’s rigour was not compromised. The researchers employed an external audit to demonstrate reliability. Transferability refers to the extent to which this study could be reproduced (Thomas, Nelson & Silverman 2011). The researchers described the methodology and process in detail to ensure the study could be replicated in other settings. Additionally, the researchers provided justification for using specific criteria for the purpose of the current study.

### Reflexibility

Undertaking qualitative research, particularly through fieldwork, transforms a researcher’s perspective. Regarding reflexivity, the researchers recognise changes experienced within themselves resulting from the research procedure and the impact these changes have on the research process (Braun & Clark 2019, 2021; Palaganas et al. 2017). The corresponding author is an advocate for people with disabilities and a researcher in leisure and disability studies. I am aware that my interpretations of participants’ experiences may be influenced by my beliefs about leisure for people with disabilities. To address this, the first author was trained as a postgraduate student to collect and analyse data and consulted with the corresponding author to promote the rigour of the study. This process supported the research process and sought to ensure transparency about how the corresponding authors’ perspectives might have influenced the study’s outcomes.

### Pilot study

Before starting data collection, a pilot study was conducted to test the research instrument and ensure it measured and collected the intended data. This includes checking whether the interview questions were clear and understandable. The pilot study involved engagement with a qualitative researcher and a topic expert to ensure the interview questions were sound. Their feedback helped improve the interview process. The goal of the pilot was to identify any problems early so that the main study could be conducted more effectively and to collect reliable information.

### Ethics considerations

Ethical clearance to conduct this study was obtained from the University of the Western Cape Humanities and Social Sciences Research Ethics Committee (HSSREC) (No. HS22/9/16). Additionally, approval was obtained from the NGO or non-profit organisation (NPO) and community leaders. A comprehensive information sheet outlining the study’s goals, participation requirements, and potential risks and benefits was provided to participants. Participants had the opportunity to ask questions once the researchers gave a verbal explanation of the study. There was a minimum 24-h to 48-h waiting period to allow individuals to decide whether to participate in the study. Participants will sign an informed consent form to demonstrate their voluntary participation, comprehension of the study, and awareness of their rights, including the right to withdraw from the study at any time without penalty. Consent from parents or legal guardians was also obtained for participants who are under guardianship or who were required to provide it. Anonymous identifiers were used to protect personal data, and no publications or reports contained data that could identify participants for at least 5 years. Data (including audio recordings and transcripts) were stored on password-protected devices before being securely erased or destroyed. All research activities complied with ethical principles of respect for individuals, beneficence, non-maleficence, and justice, in line with the Declaration of Helsinki and the university’s research ethics policy.

## Results

In this study, participants included both females and males and represented different racial backgrounds (Table 1). Some participants used wheelchairs and walking sticks. There were six out of all the 12 participants whose left side was impaired due to a stroke. Among the six participants, only three used wheelchairs because they were unable to use their lower limbs. The remaining three participants experienced balance difficulties and therefore used walking sticks for support, while another three participants had limited hand use.

**TABLE 1:** Participants’ characteristics.

Participant	Gender	Ethnic group	Age (years)	Marital status	Assistive devices
P1	Female	African person	30	Single	Wheelchair
P2	Male	Coloured person	40	Single (widower)	N/A
P3	Female	Coloured person	35	Married	N/A
P4	Female	Coloured person	37	Married	N/A
P5	Male	Coloured person	39	Married	Walking stick
P6	Male	White person	40	Single	Wheelchair
P7	Female	African person	33	Married	Walking stick
P8	Male	Coloured person	38	Married	N/A
P9	Male	Coloured person	30	Single	N/A
P10	Male	Coloured person	36	Single	Walking stick
P11	Male	African person	38	Married	Wheelchair
P12	Female	Coloured person	38	Single	N/A

N/A, not applicable.

The aim of the study was to explore and describe trends and shifts in leisure programmes for people with disabilities in the City of Cape Town municipality townships during and after the COVID-19 pandemic. The present study reports how leisure activities shifted from pre-COVID-19 to during COVID-19 to post-COVID-19; there has been a shift between the three phases. In the pre-COVID-19 period, participants reported being more actively engaged in leisure activities than during the COVID-19 pandemic, when government restrictions limited their options. The COVID-19 pandemic brought many changes to everyone’s lives, and those changes affected people’s choices of leisure activities, both with and without disabilities. Participants shared their experiences of the impact of the COVID-19 pandemic, indicating that the pandemic negatively affected their leisure participation due to the inability to access recreational hubs during lockdown. Four key themes were reported in the main study.

### Theme: Available leisure activities during the COVID-19 pandemic

Since the introduction of lockdown, the world has changed regarding how people with disabilities participate in leisure activities because they did not have the freedom of movement. The current theme supports the ICF model domains of environmental factors (specifically, the elements of support and environment) and participation, noting that during the COVID-19 pandemic, the lockdown restricted freely chosen activities. Without access to the facilities that provide leisure activities for people with disabilities, participation became challenging. Participants reported a shift in their leisure activities caused by the pandemic. This finding shows that people with disabilities can participate in activities that do not require a specific facility, depending on the type of activity and its availability. There has been a gradual shift in leisure participation during and after the COVID-19 pandemic; leisure activity preferences and trends have been constantly shifting (Kelly, Erickson & Turnnidge, 2020). In line with the ICF model in the domain of participation, choices and preferences regarding leisure programmes were particularly affected, leading to shifts and trends. Participants reported that:

‘[*During COVID-19, I was limited to doing*] Word puzzles, bingo … and the leg activities, and the arm.’ (P4, Female, Coloured person)

‘Farming is another thing. Now when I take my wheelchair and go to Stellenbosch, I sit there, I look at the birds and I look at the mountain then again you connect with God.’ (P5, Male, Coloured person)

‘Sometimes I put radio on and play my number and dance.’ (P12, Female, Coloured person)

The above reports provide a brief indication of how people had to adapt to the ‘new normal’ by adjusting and shifting their leisure activities. This was necessitated by changes brought about by the pandemic, which significantly affected participation in leisure activities. Depending on the level of autonomy offered in leisure activities, which can range from complete external control and direction to total freedom and self-direction, leisure time and spaces could be particularly helpful for improving the livelihood of people with disabilities (Lazcano et al. 2021). Participants reported that most activities were limited during the COVID-19 pandemic. These activities are viewed as passive, the participants commented, saying:

‘Activities like, dancing, watching TV and movies, and listening to the radio.’ (P12, Female, Coloured person)

‘Walking then come back and watch TV. That is how my day go.’ (P11, Male, African person)

‘[*I*] … take my grandchildren to the park and sit there for an hour or two playing.’ (P8, Male, Coloured person)

The above finding shows that people with disabilities enjoy leisure activities, but due to the lockdown, their choices were limited to passive leisure activities. It can be anticipated that the activities above may change post-COVID-19 pandemic, as the environment will be different. This sentiment falls within the ICF model, specifically within the domain of environmental factors (Hudson et al. 2022). This is regarded specifically because of the level of support and the environment in which participants found themselves during the COVID-19 pandemic. There were different lockdown levels, which varied the level of support and the environment, in turn impacting the choice of leisure activities. With the overall shift, the findings of this study show that people with disabilities made different activity choices during the COVID-19 pandemic. A study by Sivan (2020) shows that, amid restrictions, leisure activities shifted towards family-oriented activities. Additionally, Young (2020) highlighted that most activities were conducted online as technology expanded. The findings show that participants in this study engaged in passive activities such as watching TV and listening to the radio during the COVID-19 pandemic. One participant experienced some barriers during the COVID-19 pandemic, reporting that:

‘When there was COVID-19, I couldn’t go out with my friends, play bingo with them … Can’t go play soccer at the field you see.’ (P9, Male, Coloured person)

This finding shows that people with disabilities were not fully participating in active leisure activities during the COVID-19 pandemic due to changes in the types of activities available and the choices of leisure activities. This theme shows that some participants maintained the same activity choices they engaged in during the COVID-19 pandemic. In line with the broader domains of the ICF model, the current study shows that the participant was affected in three phases: Environmental factors (isolation caused by lockdown), personal factors (passive leisure rather than active programmes and limited social programmes), and health conditions (their mobility challenges).

The findings further show that after the COVID-19 pandemic, people with disabilities are not fully participating in leisure activities due to changes brought by the pandemic. They also show that participants maintained the same activities that they engaged in during the pandemic. Leisure time changed following the COVID-19 pandemic; there were common passive leisure activities and fewer people to engage in active leisure activities (Park, Lee & Kim 2020). Article 1, in the context of the study, the Charter for Leisure reports that activities such as dancing form part of active participation in social and cultural activities, which are governed by social and cultural rights, stating that every individual has the right to participate in leisure activities (Veal & Sivan 2024).

### Theme: Impact on leisure participation

Looking at how the world has changed and how people have adapted to the rise of COVID-19, there has been a shift in leisure platforms. The restrictions and regulations imposed by the COVID-19 outbreak impacted society by affecting leisure participation. In this theme, participants express how they have experienced the impact on their leisure participation through their lived experiences. This theme resonates with the ICF model domain of personal factors (Hudson et al. 2022). This accounts for the participants’ awareness of COVID-19, their knowledge of the leisure programmes and the trends caused by COVID-19, and their social function and acceptance regarding making the most of what is available. The findings from this study align with the intentions underlying participants’ behaviour in Saxena (2023). Participants shared their experiences:

‘We didn’t come to class a lot because of COVID-19 … people didn’t want us to use the church [*recreational hub*].’ (P1, Female, African person)

‘Well, it cut out most things [*COVID-19*]. You couldn’t event cycle on the road, couldn’t run on the road, walk and couldn’t go to the beach. It literally changed everything.’ (P7, Female, African person)

The experiences of leisure for people with disabilities in this theme show that leisure shifted across the pre-, during-, and post-COVID-19 periods. As a mitigation, though, this shift has allowed people to engage in leisure activities in the comfort of their homes. Additionally, Leigh (2020) reported that engaging in home-based leisure activities, such as home fitness, colouring, crocheting, and card games, was important during lockdown. In reference to the domain of personal factors under the ICF model, the element of social function is tested (Hudson et al. 2022). Participants’ reported challenges disrupt the social dynamics anticipated when engaging in freely chosen leisure activities. The COVID-19 pandemic brought significant changes to leisure activity choices due to restrictions and created opportunities for people with disabilities to improve their well-being in society:

‘So, COVID-19 is bad … But still, it impacts negatively on my leisure activities.’ (P5, Male, Coloured person)

‘Well, it cut out most things. You couldn’t even cycle on the road … couldn’t run on the road, walk and couldn’t go to the beach. It literally changed everything.’ (P6, Male, White person and P12, Female, Coloured person)

‘I couldn’t go out with my friends … Can’t play soccer at the field you see and its lockdown, I can’t go to anybody’s house was just sitting at home.’ (P9, Male, Coloured person)

The findings show that people with disabilities are passionate and involved in leisure activities; however, due to the outbreak of COVID-19, people were restricted to their homes. The findings showed that it was difficult for participants to engage in leisure activities during the COVID-19 pandemic, especially when places that offered them were closed. Lack of access to recreational hubs limited people with disabilities from engaging in leisure activities beyond their mobility. Participants’ leisure activity participation levels fell below pre-pandemic levels during the first 2 years of the pandemic (Laakso et al. 2022). A similar study by Young (2020) highlighted that the COVID-19 pandemic was a substantial obstacle to leisure participation due to restricted access to numerous leisure facilities, including sports facilities.

Contrary to the above statements, one participant shared a different comment, saying:

‘What I can tell you is that COVID-19 didn’t affect me.’ (P11, Male, African person)

Participant 11’s comment demonstrates how people were affected differently and had varied experiences during the COVID-19 pandemic. The challenges reported in this theme have a consequence on people with disabilities’ overall quality of life. This hinders the key domain in the ICF model. The ICF takes a fundamental shift from emphasising people’s disabilities to focusing on their level of health (WHO 2001). This shift aligns with the present study’s context, as it shows that all people (including those with disabilities) can achieve optimal functioning if given equal opportunities, for example, through participation in leisure activities. Considering the sentiments from Participant 11, different experiences with COVID-19 would influence optimal functioning.

### Theme: Leisure experiences

The findings in this theme report that participating in leisure activities creates a space for participants to connect. The findings show that people with disabilities enjoy leisure time, which helps them feel at ease and cope with the challenges they face in their everyday lives. Leisure activities offer the opportunity to contribute to wellbeing and increase perceptions of self-efficacy, as self-efficacy influences how people with disabilities behave and express themselves during leisure participation (Latimer & Martin Ginis 2005). This theme supports the ICF model domain, specifically the activities (Hudson et al. 2022). The participants shared the following experiences:

‘[*When I participate in leisure activities*] … I feel good, I feel relaxed. I feel good because I did get help from stroke support for more than ten years.’ (P1, Female, African person)

‘I enjoy being with other people’, ‘it makes me feel comfortable.’ (P3, Female, Coloured person)

‘*Yah*, but I enjoy myself and am happy… *Yah*, but you can’t wait to be here. We enjoy, we feel at ease, and we forget about the stroke … we were so excited.’ (P4, Female, Coloured person)

The findings of the present study indicate positive prospects, given the benefits participants gained from leisure programmes. To reiterate the point about the activity domain from the ICF model, participants in this theme report that leisure activities helped them achieve benefits they welcomed. In line with the activities from the ICF model, when leisure service providers remove limitations for participants, the level of participation increases, and people can enjoy themselves (Hudson et al. 2022). Leisure behaviour shifts across levels of participation among people with disabilities, particularly during the various stages of the COVID-19 pandemic. Adding to this notion, other participants expressed, stating the following:

‘I am at peace. Very peaceful, even sometimes get agitated by workers not doing the right thing the way it should be done.’ (P5, Male, Coloured person)

‘When you are feeling depressed, feeling that you want to lay down and sometimes you don’t want to eat … when we come here, we feel relaxed … Am happy. So, they help me believe that there’s God. They help me believe that you are old now feeling alone but you are not alone.’ (P7, Female, African person)

Participants in this study actively engaged in leisure activities through which they could express themselves. Participants can enjoy the benefits of being actively involved in leisure activities if limitations and restrictions are removed, in line with the ICF model, to facilitate active participation. Hudson et al. (2022) explained that people with disabilities can enjoy participating in activities when mechanisms are in place to reduce the barriers they encounter, aligning with the ICF model’s domains of activities and personal factors. Another participant reported:

‘… feeling good when we are together with their peers, participating in leisure programs offered by the recreational centre … [*My*] mood is lifted every time after the [*leisure*] program.’ (P3, Female, Coloured person)

One participant shared a valuable lesson that they now use as motivation. This notion is aligned with Malema et al. (2024), who argued that people found it difficult to adjust to the major changes in daily life brought about by the COVID-19 pandemic. The findings from this study revealed that participating in leisure activities provides a space to build relationships and connect with other people. A study by Byrne et al. (2006) argued that leisure time provides opportunities to try out social roles, identify personal preferences, exchange experiences, develop teamwork skills to deal with various situations, and form friendships. The participant reports that:

‘[*Throughout being involved in leisure activities*] I never take these things [*leisure programmes*] for granted, frankly, personally I have learned to love people that I associate with.’ (P2, Male, Coloured person)

Every individual has the right to engage in leisure activities, regardless of their age or living conditions. The findings of the present study show that people with disabilities value the opportunity for leisure programmes. These findings align with the environmental and personal factors in the ICF domain (WHO 2023). The COVID-19 pandemic and the lockdown regulations impacted the shift and decline in leisure activities for all citizens, especially for people with disabilities who were already sidelined from mainstream activities. A study by Berg, Warmer and Das (2015) expressed that changes in technology, the economy, society and the built environment contributed to declining rates of physical activity. As a result of the decline in physical activity, more sedentary activities, especially those involving ‘screen time’, such as playing video games, using tablets, using handheld gaming consoles, using computers, and watching television, are becoming more popular.

### Theme: Future prospects of leisure participation

This theme examines how people perceive leisure participation, which influences their preparation and willingness to engage in it as they seek the benefits associated with leisure activities. The findings show that people with disabilities enjoy their leisure time and participation in leisure activities. Moreover, they are always eager to participate in these activities. With reference to the ICF model, the current theme report focuses on activity-related elements that cut across the domains of environmental factors and participation (Hudson et al. 2022). This shows that they view leisure activities as important for the future and prepare themselves to use these opportunities to improve and maintain their physical and psychological health. Participants expressed their perceptions on their future participation:

‘Now I want to exercise especially at home … but I need a right partner to exercise with.’ (P1, Female, African person)

‘I continually see myself in a more fluent moving condition … as far as am concerned.’ (P2, Male, Coloured person)

‘I will do it as long as I can.’ (P11, Male, African person)

The findings of this study showed that people with disabilities enjoy participating in leisure activities and always look forward to their leisure time. Changes in attitudes towards people with disabilities can make them more likely to say that physical activity is suitable for people like them (Limbpower 2021). The sentiments above are supported by domain participation within the ICF model, as evidenced by the findings of the study. The key issue is removing barriers that could limit or restrict people with disabilities from freely participating in leisure activities. Therefore, the barriers within their leisure spaces, particularly the temporal climate created by COVID-19, can be negotiated as leisure service providers adjust their program offerings to accommodate environmental changes. This is further expressed by other participants, reporting that:

‘I want to come here [*recreational hub*]. I want to dance more.’ (P3, Female, Coloured person)

‘I see the future. I am very positive about that. Really, it is something good.’ (P4, Female, Coloured person)

Other participants reported that:

‘Future prospects are positive considering the benefits we get when engaged in the [*leisure*] programmes.’ (P8, Male, Coloured person)

In light of the future prospects for leisure activities, it is anticipated that passive leisure activities will no longer be as prevalent, considering that the lockdown regulations are no longer in effect. This means that the freely chosen activities would revert to the pre-COVID-19 era. Participation in leisure activities provides opportunities for health improvement and well-being and helps build relationships with other people in the community. The findings of the current study are aligned with those of Aitchison (2003), which highlight that leisure experiences enhance the quality of life, helping people build and maintain relationships with family and friends, relieve tensions and make their lives more bearable. Leisure behaviour is determined by a person’s intentions towards leisure activities (Hutzler & Korsensky 2010; Latimer & Martin Ginis 2005). Leisure plays a crucial role in the lives of people with disabilities, providing them with space to connect with others, freedom of action, and the ability to express themselves.

### Implications for leisure in communities

The findings of this study reported that, because of the COVID-19 pandemic, the choices and preferences for leisure activities shifted from active leisure to passive leisure among people with disabilities. The key findings from the present study reported that people felt calm and at ease despite the COVID-19 pandemic because leisure offers a way to temporarily forget their living circumstances. The community and organisations supporting people with disabilities should work together to ensure these individuals feel included. There is a need for local municipalities to upgrade and maintain facilities that offer leisure activities for people with physical disabilities, ensuring easier access to leisure spaces. This is important because the findings show that most of the leisure activities that people with disabilities participate in are influenced by their physical abilities and their limitations due to the environment they live in.

These regular upgrades and maintenance could significantly increase participation in leisure activities among people with disabilities. The community and organisations supporting people with disabilities should work together to ensure these individuals feel included in community life during leisure activities. People with disabilities value the opportunities they have, and their knowledge of the benefits of participating in leisure activities influences the way they express themselves through their behaviour. Participants’ behaviour is positive, indicating they see value in the leisure activities they engage in.

### Limitation of the present study

The present study acknowledges that it is not without limitations. The use of purposive sampling and inclusion criteria posed a limitation for the present study. The researchers acknowledge that the inclusion of adults with physical disabilities may limit the transferability of the present study. Despite the reported limitations, the findings of the present study offer significant insight into current research and make a substantial contribution to the literature on this topic.

## Conclusion

The researchers conclude that people with disabilities have their preferences for leisure activities according to their physical abilities. The researchers also conclude that people with disabilities are well aware of the benefits of participating in leisure activities. Participation in leisure activities helps people with disabilities to not focus on their limitations; rather, they enjoy leisure activities because it improves their movement. This study concludes that leisure activity participation for people with disabilities was affected by the COVID-19 pandemic, which shifted the choices of leisure activities as a result of the pandemic. The COVID-19 pandemic had a psychological and physical impact on participants and shifted their level of participation in leisure activities.
